# Castleman Disease in Tunisia: A Multicenter Clinical, Prognostic, and Therapeutic Study

**DOI:** 10.1155/ah/1623836

**Published:** 2026-09-20

**Authors:** Tayssir Ben Achour, Syrine Sassi, Aida Khadar, Wafa Skouri, Mariem Borgi, Maysem Jridi, Arfaoui Bilel, Aydi Zohra, Adaily Najeh, Amri Raja, Ben Abdelhafidh Nadia, Ben Abdelghni Khaoula, Ben Fraj Fatma, Ben Hssine Lamia, Boussema Fatma, Daada Syrine, Guediche Nour El Houda, Ines Chelly, Jebri Meriem, Smiti Monia

**Affiliations:** ^1^ Department of Internal Medicine, Rabta Hospital, Tunis, Tunisia; ^2^ Faculty of Medicine of Tunis, Tunis El Manar University, Tunis, Tunisia, utm.rnu.tn; ^3^ Department of Internal Medicine, Charles Nicolle Hospital, Tunis, Tunisia, chucharlesnicolle.tn; ^4^ Departement of Anatomical Pathology, Rabta Hospital, Tunis, Tunisia; ^5^ Department of Internal Medicine, Mahmoud Matri Hospital, Nabeul, Tunisia; ^6^ Department of Internal Medicine, Military Hospital, Bizerte, Tunisia; ^7^ Department of Internal Medicine, Habib Thameur Hospital, Tunis, Tunisia, hopital-h-thameur.rns.tn; ^8^ Department of Internal Medicine, Farhat Hached Hospital, Sousse, Tunisia, chu-hached.rns.tn; ^9^ Department of Internal Medicine, Miltary Hospital, Tunis, Tunisia; ^10^ Department of Internal Medicine, Sahloul Hospital, Sousse, Tunisia

**Keywords:** Castleman disease, HHV8, multicentric, unicentric

## Abstract

**Introduction:**

Castleman disease is an uncommon benign lymphoproliferative condition that includes diverse clinical, biochemical, and histological variations, each characterized by unique prognoses and therapeutic approaches. The aim of our study was to characterize the clinical and paraclinical features, as well as the management approaches, of Castleman disease in the Tunisian population.

**Methods:**

We conducted a multicenter study including patients with a histologically confirmed diagnosis of Castleman disease who were followed in the Internal Medicine departments of various hospitals across Tunisia between 2000 and 2024.

**Results:**

Sixteen patients (55.2%) with multicentric Castleman disease and 13 patients (44.8%) with the unicentric form were included. The most frequent initial manifestations were lymphadenopathy (69%), mass (20%), fever (26.7%), and weight loss and fatigue (56.7%). HHV‐8 tested positive in 10.7%, while 3.6% of patients had autoantibodies. Hyaline‐vascular (53.6%), plasmacytic (32.1%), and mixed (14.3%) subtypes were diagnosed, with a distinct plasmacytic predominance among multicentric types. Following total surgical resection, 14 patients (48.3%) experienced immediate radiological and clinical remission. On the other hand, systemic therapy was required for patients with multicentric types. In total, 12 patients (44.4%) experienced full remission, (14.8%) remained stable, and (3.7%) continued to progress despite treatment.

**Conclusion:**

With a significant percentage of multicentric forms and a low HHV‐8 seropositivity rate, our Tunisian study validates the various manifestations of Castleman disease.

**Highlights**
• Although it is frequently underdiagnosed, Castleman illness is a reality in Tunisia.
• The majority are multicentric types having a systemic and inflammatory profile.
• Future management paradigms are expected to emphasize targeted therapies alongside comprehensive multidisciplinary collaboration.

## 1. Introduction

Castleman disease (CD) or angiofollicular lymphoid hyperplasia, is a rare benign lymphoproliferative disorder characterized by uni‐ or multicentric lymph node enlargement caused by polyclonal lymphoid proliferation. It encompasses distinct clinical, biological, and pathophysiological entities [1].

The etiopathogeny of this disease remains unknown, and its clinical presentation is often nonspecific. Benjamin Castleman initially identified it as asymptomatic mediastinal masses with distinctive “hyaline‐vascular” histological characteristics in 1956 [2, 3]. Since that initial report, the disease’s clinical and pathological spectrum has broadened. Multicentric forms with significant systemic and biological involvement have been reported [4, 5].

CD may affect everyone at any age, even young children [6]. Often asymptomatic, its diagnosis remains a significant problem in clinical practice. A close collaboration between internists, pathologists, and radiologists is essential to establish this often elusive diagnosis.

Our study’s objective was to characterize the clinical and paraclinical aspects of CD in the Tunisian population, as well as the management measures.

## 2. Methods

This multicenter retrospective study was conducted across several tertiary medical centers in Tunisia between 2000 and 2024.

### 2.1. Study Population

Inclusion Criteria:

Histologically confirmed diagnosis of CD.

Tunisian Nationality:

Complete clinical records available for analysis.

Noninclusion Criteria:

Patients whose ‘Castleman‐like’ histological features could be attributed to another underlying medical condition (e.g., infectious, autoimmune, etc.)

Exclusion Criteria:

Patients whose medical records were missing and/or not usable.

### 2.2. Data Collection

A standardized case report form was used to gather data retrospectively from patient medical records. Demographic data (age and gender), clinical presentation and symptoms at diagnosis, laboratory parameters (inflammatory markers and virological status, including HHV‐8 and HIV), imaging findings, histological subtype, treatment modalities (surgery, corticosteroids, immunosuppressants, anti‐IL‐6 therapy, and chemotherapy), and disease course and outcomes were analyzed.

### 2.3. Ethical Considerations

The ethics committee approved the study protocol. Given the retrospective nature of the study, informed consent was waived according to the local requirements. Patient data were anonymized before analysis.

### 2.4. Statistical Analysis

Descriptive statistics were used to summarize patient characteristics. Categorical variables were expressed as frequencies and percentages, while continuous variables were described using means ± standard deviation or medians with interquartile ranges, as appropriate. Comparisons between subgroups were conducted using chi‐square or Fisher’s exact test for categorical variables and Student’s *t*‐test or Mann–Whitney *U* test for continuous variables. A *p* value < 0.05 was considered statistically significant.

## 3. Results

### 3.1. Clinical and Epidemiological Characteristics of the Study Population

The study included 29 patients with a mean age of 40 years and a sex ratio of 1.15 (53.6% female). The mean age at diagnosis was 43. Seven months was the mean diagnostic delay.

### 3.2. Clinical and Paraclinical Characteristics

Sixteen patients (55.2%) presented with multicentric CD and 13 patients (44.8%) with the unicentric form.

Among the MCD cases, the majority were classified as idiopathic multicentric CD (iMCD). There were no cases fulfilling the criteria for iMCD‐TAFRO. The remaining iMCD cases were therefore classified as iMCD‐NOS. In addition, 10.7% of the entire cohort (3 patients) had HHV‐8‐associated MCD. None of the patients with HHV‐8‐associated MCD had HIV infection. No cases of POEMS‐associated MCD were identified in our cohort.

Regarding UCD, none of the patients presented with systemic inflammation, paraneoplastic pemphigus, bronchiolitis obliterans, or AA amyloidosis.

The most frequent initial manifestations were lymphadenopathy (69%), mass (20%), fever (26.7%), weight loss, and fatigue (56.7%).

Inflammatory markers were elevated in 51.7% of cases. No patient tested positive for HIV; 10.7% of patients tested positive for HHV‐8; 3.6% of patients had autoantibodies. The clinical and biological data are detailed in Table 1.

**TABLE 1:** Clinical and biological characteristics of patients with unicentric and multicentric Castleman disease.

Clinical and laboratory characteristics	UCD (*n* = 13)	MCD (*n* = 16)
Mean age (years)	45	55
Ethnicity	Tunisian	Tunisian
Low hemoglobin	6	11
Thrombocytopenia	4	10
Elevated CRP	10	16
Hypoalbuminemia	1	5

**FIGURE 1:**
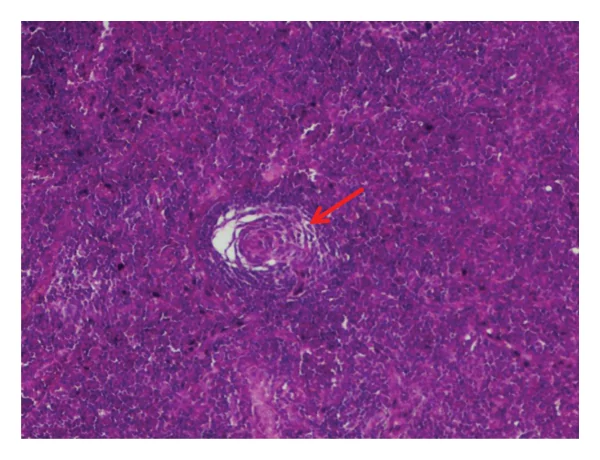
Histological section showing the hyaline‐vascular subtype of Castleman disease. Small lymphoid follicle with atrophic germinal centers surrounded by concentric layers of mantle zone lymphocytes. H&E stain × 100.

**FIGURE 2:**
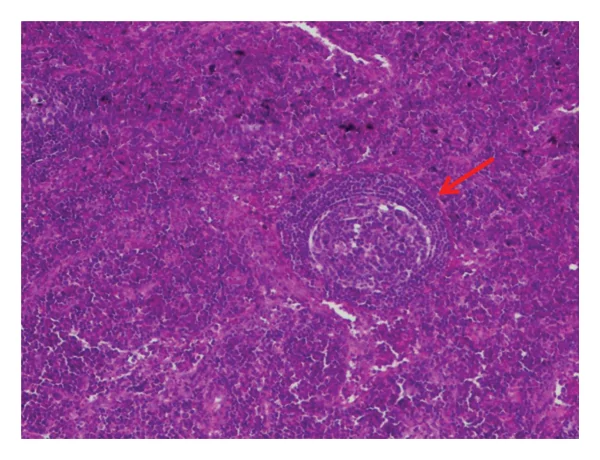
Histological appearance of the concentric mantle zones (‘onion‐skinning’). H&E stain × 100.

**FIGURE 3:**
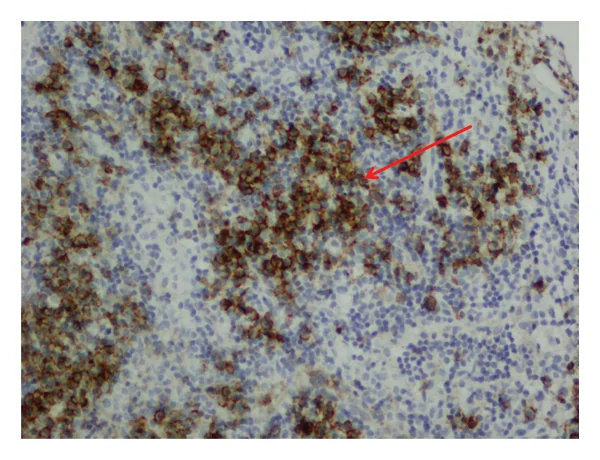
Immunohistochemical staining highlighting sheets of mature plasma cells in the interfollicular regions, characteristic of the plasma cell variant of Castleman disease.

Histopathology revealed a distribution of hyaline‐vascular (53.6%), plasmacytic (32.1%), and mixed (14.3%) subtypes, with a clear plasmacytic predominance among multicentric cases (Figures 1, 2, and 3).

### 3.3. Therapeutic Modalities

The two clinical phenotypes had rather different treatment approaches. Following total surgical resection, 14 patients (48.3%) achieved immediate radiological and clinical remission. On the other hand, systemic therapy was required for individuals with multicentric disease. Twelve patients (42.9%) received first‐line corticosteroids, seven (25%) received anti‐IL‐6 therapy (tocilizumab 8 mg/kg), and three (10.7%) of the refractory patients had multiagent chemotherapy with R‐CHOP (rituximab, cyclophosphamide, doxorubicin, vincristine, and prednisone) administered. In total, 12 patients (44.4%) experienced full remission, 14.8% remained stable, and 3.7% continued progress despite treatment.

## 4. Discussion

The epidemiological, clinical, paraclinical, therapeutic, and prognostic features of Tunisian patients with CD followed in six internal medicine departments could be described due to our study.

CD is a rare condition. According to worldwide studies [7, 8], the unicentric form (UCD) is generally more common than the multicentric form (MCD), resulting in roughly 70%–80% of the cases. However, MCD accounted for 56.7% of cases in our cohort, suggesting either a bias in recruitment toward more severe cases treated in academic medical centers or a distinct distribution in our population. According to Hoffmann et al.’s findings [9], our patients’ mean age was 45.5 years, with a slight female predominance.

In our study, 7 months was the mean diagnostic delay. This delay for multicentric CD is long but not unusual, as most patients are initially misdiagnosed and treated as tuberculosis, which is more prevalent in our country, and occasionally as Kaposi’s sarcoma. It also is explained by the difficulty in accessing a biopsy for histological confirmation. The diagnostic delay usually leads to more advanced disease and poorer outcomes in these patients.

Exaggerated immunological activation is a key component of pathophysiology, with HHV‐8 in HIV‐associated cases and interleukin‐6 (IL‐6) in idiopathic forms. The majority of the patients in our study were HIV‐negative.According to the CD classification proposed by Chen et al. [10]. None of the patients tested positive for HIV, and only 10.7% of them tested positive for HHV‐8, ruling out a viral etiology. These results strengthen the hypothesis of an idiopathic inflammatory origin in our population, in line with the observations of Yu et al. [11] and Fajgenbaum et al. [7]. The absence of HIV‐positive cases can be explained by several factors such as Tunisia having a significantly lower national HIV prevalence compared to many sub‐Saharan African countries. In our cohort, systematic HIV testing was performed, and indeed none of the patients tested positive. Moreover, differences in referral patterns and epidemiological backgrounds may also help explain this finding.

Clinically, asthenia, weight loss (56.7%), and fever (26.7%) were common systemic manifestations. These symptoms are linked to an excess of proinflammatory cytokines and are usually reported in multicentric forms [7, 8].

More than half of the patients (51.7%) had an inflammatory syndrome, and their biochemical abnormalities (elevated CRP, anemia) were consistent with immune activation. According to published data [7], the plasmacytic subtype (32.1%) predominated in MCD, whereas the hyaline‐vascular subtype (53.6%) predominated in UCD.

The predominance of the hyaline vascular subtype (53.6%) in our cohort differs from several studies in sub‐Saharan Africa, where plasma cell or mixed variants are more common, often in association with HIV/HHV‐8 infection. We believe this discrepancy may be multifactorial. The absence of HIV‐positive patients in our cohort likely influenced the histological distribution, as HIV‐associated CD is more frequently of the plasma cell type. In addition, geographic and epidemiologic variations in HHV‐8 prevalence may contribute, and potential local genetic or environmental factors cannot be excluded. Differences in case selection, such as the proportions of unicentric versus multicentric cases, may also partially explain this variation.

According to Boutboul et al. [12], surgical resection was the primary treatment for UCD, and it was curative in 84.6% of cases with a 100% overall survival rate [7]. In contrast, systemic therapy was needed for MCD. Three patients in our group experienced total remission following first‐line corticosteroid therapy. Nevertheless, a number of patients experienced corticosteroid resistance, requiring the addition of anti‐IL‐6 biotherapy or chemotherapy. Our results are comparable to those of Van et al. [13] and Zhang et al. [14].

While MCD has a less favorable prognosis, UCD has an excellent prognosis. Four deaths in our series happened in the MCD group as a result of multiorgan failure or infectious complications. In line with the findings of Zhang et al. [14], this mortality rate underlines the severity of systemic forms.

We were able to examine the epidemiological, clinical, and therapeutic features of CD in Tunisia due to the multicentric design that included multiple tertiary hospitals. The study’s retrospective design remains a limitation, though.

## 5. Conclusion

Our Tunisian series confirms the heterogeneous presentations of CD, with a high proportion of multicentric forms and a low HHV‐8 seropositivity rate. It highlights the importance of tailoring management to the clinical phenotype and the potential benefit of IL‐6–targeted therapies. Larger prospective studies are warranted to improve diagnostic and therapeutic strategies in our context. This article contributes to alerting pathologists and clinicians to consider the diagnosis sooner rather than later. Earlier diagnosis has to be a goal we achieve as quickly as possible

## Author Contributions

All authors contributed to the study conception and design. Tayssir Ben Achour, Syrine Sassi, Wafa Skouri, and Mariem Borgi collected and analyzed the data and drafted the manuscript. Tayssir Ben Achour had full access to all of the data in this study and takes complete responsibility for the integrity of the data and the accuracy of the data analysis. All authors critically revised the manuscript and contributed to the interpretation of the results.

## Funding

The authors have nothing to report.

## Disclosure

All authors approved the final version of the manuscript.

## Ethics Statement

This study was conducted in accordance with the Declaration of Helsinki. Ethical approval was obtained from the appropriate ethics committee of Rabta Hospital.

## Consent

Written informed consent was obtained from all patients for the publication of their clinical data and images.

## Conflicts of Interest

The authors declare no conflicts of interest.

## Data Availability

The data supporting the findings of this study are available from the corresponding author upon reasonable request. Due to ethical and privacy considerations, the data are not publicly available.
